# Triglyceride-glucose index, HOmeostatic Model Assessment index, and new-onset hypertension in middle-aged men

**DOI:** 10.1097/HJH.0000000000004162

**Published:** 2025-09-24

**Authors:** Lanfranco D'Elia, Domenico Rendina, Roberto Iacone, Ornella Russo, Pasquale Strazzullo, Ferruccio Galletti

**Affiliations:** Department of Clinical Medicine and Surgery, ESH Excellence Centre of Hypertension, “Federico II” University of Naples Medical School, Naples, Italy

**Keywords:** blood pressure, HOmeostatic Model Assessment of Insulin Resistance index, hypertension, insulin resistance, triglyceride-glucose index

## Abstract

**Objective:**

Hypertension is a major risk factor for cardiovascular diseases. Insulin resistance is one of the main risk factor for hypertension. A simple index (triglyceride-glucose index – TyG) has been considered as a surrogate marker of insulin resistance. Although several studies have explored TyG and cardiovascular risk, few longitudinal data on the relationship between new-onset hypertension and this novel index are available so far, especially in European countries. Therefore, we aimed to estimate the predictive role of TyG, in comparison to that of the HOmeostatic Model Assessment of Insulin Resistance (HOMA-IR) index (a widely used tool to assess insulin resistance), on the development of hypertension, in an 8-year follow-up observation of a sample of adult men.

**Methods:**

The analysis included 482 men (The Olivetti Heart Study), without hypertension at baseline. The optimal cut-off point of the association of continuous TyG or HOMA-IR index with new-onset hypertension was identified by receiver-operating characteristic (ROC) analysis.

**Results:**

TyG was linearly associated with the occurrence of new-onset hypertension, whereas HOMA-IR was nonlinearly related to the risk of developing hypertension. After stratification by the optimal cut-off point, TyG greater than 4.91 were significantly associated with new-onset hypertension, also after adjustment for main confounders. In contrast, the HOMA-IR index greater than 1.82 was not associated with the risk of new-onset hypertension in the adjusted models.

**Conclusion:**

The principal findings of this study suggest that the TyG index exhibits a significant predictive capacity for the development of new-onset hypertension. Although its limited sensitivity, the results support the potential utility of TyG as a simple, cost-effective, and noninvasive adjunctive tool for the early assessment of cardiovascular risk.

## INTRODUCTION

Hypertension is a major risk factor for cardiovascular diseases [1,2], and approximately 1.28 billion adults worldwide (30–79 years) have hypertension [1]. Several cardiometabolic factors are considered modifiable risk factors associated with hypertension, among which insulin resistance is a major one [2,3]. Indeed, insulin resistance can affect cardiovascular risk through impairment of the nitric oxide (NO) pathway [4], activation of the sympathetic nervous system [5], and alterations in renal sodium handling and fluid retention [3,6]. Thus, the early identification of individuals with insulin resistance plays a key role in classifying individuals at high cardiovascular risk. Many markers have been tested to assess insulin resistance; the hyperinsulinaemic–euglycemic clamp is considered the gold standard technique [7], but its complexity does not allow its use in clinical practice. Among all the surrogate tools, the Homeostatic Model Assessment of insulin resistance (HOMA-IR) index is currently the most widely used insulin resistance marker in epidemiological studies and clinical practice [7]. However, the use of insulin level measurement for assessment limits its use in clinical practice and for experimental and observational studies on large samples for obvious reasons. In recent years, a novel index has been proposed to address these limitations: the triglyceride-glucose index (TyG), which is simple, convenient, and inexpensive, because it does not require insulin measurement [8,9]. Both TyG and HOMA-IR have a good correlation with the hyperinsulinaemic–euglycemic clamp and with each other [10]; however, it is important to note that TyG reflects liver and peripheral insulin resistance, whereas HOMA-IR specifically measures insulin resistance in the liver [11].

Epidemiological studies have suggested a substantial direct association between TyG and cardiovascular risk in the general populations [11–16] and cohorts at high cardiovascular risk [11,12,17]; although some studies have reported conflicting results [18,19]. TyG is also positively associated with hypertension [20,21], but with little and heterogeneous longitudinal data, primarily due to different study designs (i.e. prospective, retrospective, and cross-sectional) and characteristics (e.g. sample size, length of follow-up, and age). Moreover, only one study was conducted in Europe [22], without an assessment of the shape of the association between TyG and the risk of hypertension.

Therefore, considering that one of the global targets for noncommunicable diseases is to reduce the prevalence of hypertension by 33% between 2010 and 2030 [1], given the crucial role of early markers of the risk of hypertension to achieve this target and the limited epidemiological evidence on the relationship between TyG and the development of hypertension in European populations [11,20,21], we explored the predictive role of baseline TyG in the development of hypertension in an 8-year follow-up study of an unselected sample of men participating in the Olivetti Heart Study (OHS). In particular, the shape of the association between TyG and new-onset hypertension was assessed, and, in addition, the predictive powers of TyG and HOMA-IR were compared.

## METHODS

### Study population

The OHS was an occupational investigation of a male workforce in Southern Italy [23,24]. This study was planned, conducted, and reported in accordance with the STrengthening the Reporting of OBservational studies in Epidemiology (STROBE) statement (https://www.equator-network.org/reporting-guidelines/strobe/; accessed 10 October 2024) (Supplemental Table 1). A total of 1085 individuals (aged between 25 and 75 years) were examined in 1994–1995, and 84% were seen again in 2002–2004. For the present analysis, we sequentially excluded participants: without a complete database in both examinations (*n* = 274); and those with a diagnosis of hypertension at baseline (systolic blood pressure - SBP or diastolic blood pressure - DBP ≥140 or 90 mmHg or antihypertensive therapy) (*n* = 329). Finally, 482 participants were included in the analysis.

The Ethics Committee of ‘Federico II’ University in Naples approved the study protocol, and the participants provided their informed written consent to participate.

### Examination procedures

The OHS procedures have been previously described [23,24]. The baseline and follow-up visits included anthropometric measurements, physical examination, blood tests, and administration of a questionnaire.

Body weight and height were measured by using a standard beam balance scale with an attached ruler. BMI was measured according to the formula: weight (kg)/height^2^ (m), in particular, excess body weight was defined as BMI at least 25 kg/m^2^. Waist circumference was measured at the level of the umbilicus with the subject standing erect with a flexible inextensible plastic tape. Abdominal obesity was defined as a waist circumference greater than 102 cm.

SBP and DBP were measured three times at 2-min intervals, with a random zero sphygmomanometer (Gelman Hawksley Ltd., Sussex, UK) after the subject had been sitting for at least 10 min. The average values of the second and third readings were recorded. Hypertension was defined as a SBP greater than 140 mmHg or a DBP greater than 90 mmHg, or antihypertensive treatment [2].

Fasting venous blood samples were also obtained. Blood specimens were immediately centrifuged and stored at −70 °C until analysis. Blood glucose and triglyceride levels were measured using automated methods (Cobas-Mira, Roche, Italy). The TyG index was calculated using the following formula: Ln [TG (mg/dl) × fasting glucose (mg/dl)]/2 [8]. Blood insulin levels were determined using radioimmunoassay (Insulin Lisophase; Technogenetics, Milan, Italy). HOMA-IR was calculated using the following formula: [fasting plasma insulin (μU/ml) × fasting plasma glucose (mmol/l)/22.5]. Serum creatinine levels were measured using the picric acid colorimetric method. The estimated glomerular filtration rate (eGFR) was calculated by standard formula using the Chronic Kidney Disease Epidemiology Collaboration (CKD-EPI) 2009 equation [25]. C-reactive protein (CRP) levels were assessed using an immunoturbidimetric method (Roche Diagnostics, Milan, Italy, automated analyzer).

Participants were classified into two groups according to alcohol consumption: at least one glass of wine (or an equivalent amount of other alcoholic beverages per day) and no alcohol consumption. Physical activity level was expressed according to whether the participant habitually engaged in at least 30 min per day of aerobic exercise. Baseline smoking status was investigated using a questionnaire that classified the participants into current smokers, never smokers, and ex-smokers.

### Statistical analysis

All statistical analyses were performed using the SPSS software (version 29, SPSS Inc., Chicago, Illinois, USA) and the statistical package R (version 4.3.1). As baseline HOMA-IR, eGFR, glucose, triglyceride, and CRP levels had a skewed distribution, log-transformed values were used for the analyses. Furthermore, baseline SBP and DBP were stratified into tertiles to include them as a covariate in the multivariate analyses. Bivariate relationships between TyG, HOMA-IR, and the variables under investigation were evaluated using Pearson's correlation analysis. The analysis of variance was used to assess differences between group means, whereas the chi-square test was used to estimate differences between categorical variables. Restricted cubic splines (RCS) regression models with four knots (5th-reference, 35th, 65th, and 95th percentiles) were utilized to estimate the type of association between TyG or HOMA-IR (as a continuous variable) and the risk of new-onset hypertension [26]. Given the linear relationship between TyG and new-onset hypertension, binary logistic regression analysis was used to estimate the predictive role of baseline TyG (as a continuous variable) in the development of hypertension. The impact of traditional risk factors and that of potential confounding factors of the sample (*P* value <0.2, relating to the comparison between those who developed hypertension and those who did not – Supplemental Table 2) was explored using multivariate models adjusted for baseline age, body weight or abdominal obesity, SBP or DBP, cigarette smoking, diabetes status, and physical activity.

Receiver-operating characteristic (ROC) analysis was carried out, and the area under the curve (AUC), with its 95% confidence interval (CI), was calculated to compare the ability of TyG and HOMA-IR to identify participants who would develop hypertension at follow-up. Next, the optimal cut-off point (Youden's criterion) for the association of continuous TyG or HOMA-IR with new-onset hypertension was identified by ROC analysis (TyG: 4.91; HOMA index: 1.82). Based on these cut-off points, the sample was stratified by two groups both for TyG (high-TyG: TyG >4.91; low-TyG: TyG ≤4.91) and HOMA-IR (high-HOMA-IR: HOMA-IR >1.82; low-HOMA-IR: HOMA-IR ≤1.82), and separately analysed. Binary logistic regression analysis was used to estimate the role of baseline TyG or HOMA-IR (both as dichotomous variables) on hypertension risk, adjusting for the main potential confounders (see above).

The results are reported as percentages, as mean with standard deviation (SD) or as odds ratio (OR) and 95% CI (Bootstrap CI, 1000 iterations). Two-sided *P* values less than 0.05 were considered statistically significant.

## RESULTS

The baseline characteristics of the total sample (*N* = 482) are reported in Table 1. At baseline, TyG was significantly and directly correlated with HOMA-IR (*r* = 0.28), BMI (*r* = 0.20), waist circumference (*r* = 0.19), CRP (*r* = 0.16), and age (*r* = 0.09), and inversely correlated with renal function (*r* = −0.13). By contrast, no correlation was found with SBP and DBP (*P* > 0.05).

**TABLE 1:** Baseline characteristics of the study participants

Number of participants	482
Age (years)	50.0 (6.7)
BMI (kg/m^2^)	26.4 (2.8)
Normal weight (%)	30.4
Overweight (%)	58.8
Obesity (%)	10.8
Waist circumference (cm)	92.7 (8.0)
Abdominal obesity (%)	10.3
Systolic BP (mmHg)	119.9 (10.1)
Diastolic BP (mmHg)	78.6 (6.6)
eGFR (ml/min/1.73 m^2^)^a^	97.7 (1.2)
Renal damage (eGFR <60 ml/min/1.73 m^2^) (%)	0.6
Blood triglycerides (mg/dl)^a^	131.8 (1.7)
Blood glucose (mg/dl)^a^	97.4 (1.2)
HOMA-IR (units)^a^	1.82 (1.70)
Diabetes (%)	5.4
TyG (units)	4.74 (0.27)
Lipid-lowering therapy (%)	11
C-reactive protein (mg/l)^a^^,^^b^	1.11 (2.57)
Smoking	
Never smokers	15.4
Current smokers	51.7
Former smokers	33.0
Physical activity – yes (%)	33.1
Alcohol consumption – yes (%)	82.2

Data are expressed as means (SD) or as percentages. BP, blood pressure; eGFR, estimated glomerular filtration rate; HOMA-IR, homeostatic model assessment of insulin resistance index; TyG, triglyceride-glucose index.

a Data expressed as geometric mean.

b *n* = 436

At the 8-year follow-up, the overall new-onset hypertension rate was 53.7%. Baseline TyG and HOMA-IR were significantly greater in those who developed hypertension at follow-up than in those who did not (TyG, 4.77 ± 0.27 vs. 4.69 ± 0.28, *P* = 0.002; HOMA-IR, 1.90 ± 1.78 vs. 1.70 ± 1.62, *P* = 0.01).

**FIGURE 1:**
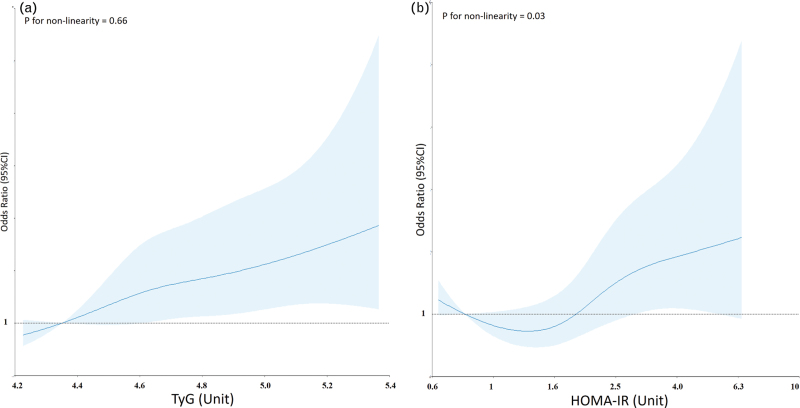
Association between the triglyceride-glucose (TyG) index or HOmeostatic Model Assessment of Insulin Resistance (HOMA-IR) index and the risk of new-onset hypertension using a Restricted Cubic Spline Regression Model. Solid lines indicate odds ratios (ORs), and shadow shapes indicate 95% confidence intervals (CIs).

The RCS regression model detected a linear relationship between TyG and new-onset hypertension (test for nonlinearity: *P* = 0.66) (Fig. 1a). Logistic regression analysis showed a significant association between baseline TyG and the risk of new-onset hypertension at follow-up (for a 1-unit increase in baseline TyG, OR: 2.83, 95% CI: 1.43–5.60). The significant predictive role of TyG was confirmed upon adjustment for the main confounders (for a 1-unit increase in TyG: model including excess body weight, OR: 2.30, 95% CI: 1.06–4.98; model including abdominal obesity, OR: 2.33, 95% CI: 1.08–5.02). By contrast, the RCS regression model showed a nonlinear association between HOMA-IR and new-onset hypertension (test for nonlinearity: *P* = 0.03) (Fig. 1b).

**FIGURE 2:**
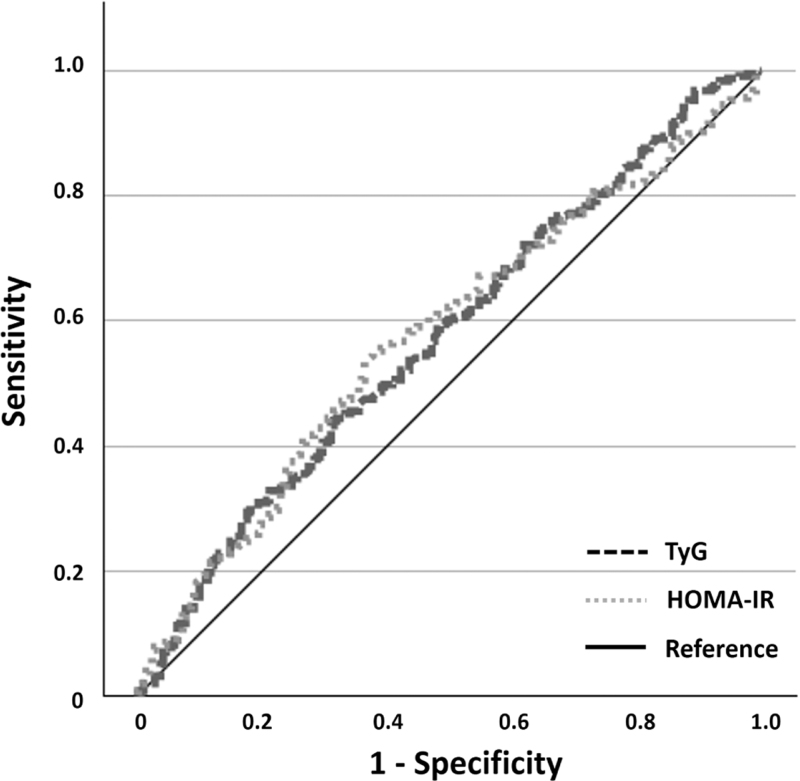
Receiver-operating characteristic curves for the triglyceride-glucose (TyG) index and HOmeostatic Model Assessment of Insulin Resistance (HOMA-IR) index.

The AUC for the relationship between TyG and new-onset hypertension showed a significant ability to detect hypertension (AUC and asymptotic 95% CI: 0.57, 0.52–0.63, *P* = 0.004; sensitivity: 30%, specificity: 82%; positive predictive value: 70%; negative predictive value: 51%); likewise, the AUC of HOMA-IR showed similar values (AUC and asymptotic 95% CI: 0.58, 0.53–0.63, *P* = 0.003; sensitivity: 56%, specificity: 60%; positive predictive value: 62%; negative predictive value: 54%). As expected, the comparison of the AUCs revealed no statistically significant difference between the two indices (*P* = 0.95; Fig. 2), indicating that TyG and HOMA-IR exhibit comparable discriminatory abilities in predicting new-onset hypertension.

**FIGURE 3:**
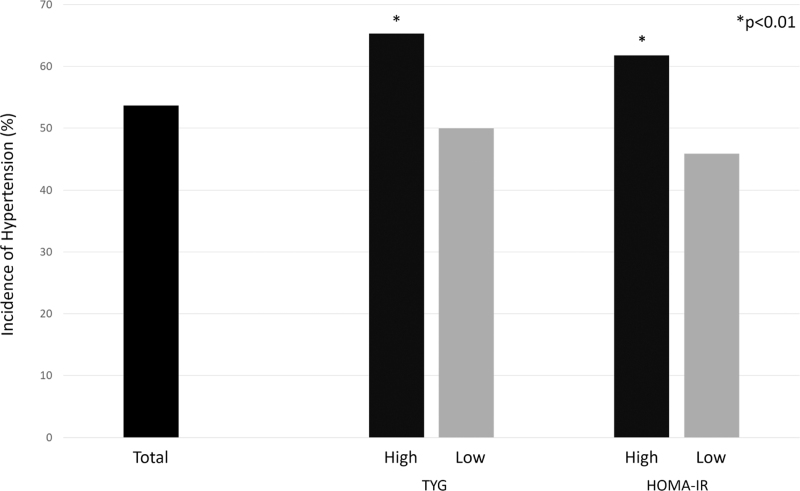
The incidence rate of new-onset hypertension in total and stratified by the triglyceride-glucose index and HOmeostatic Model Assessment of Insulin Resistance. High-TyG: >4.91, low-TyG: ≤ 4.91; high-HOMA-IR: >1.82, low-HOMA-IR: ≤1.82. ^*^High vs Low: <0.01. HOMA-IR, HOmeostatic Model Assessment of Insulin Resistance; TYG, triglyceride-glucose index.

Based on stratification using the optimal cut-off points identified by ROC curve analysis, we separately assessed the predictive value of these thresholds for both TyG and HOMA-IR. As expected, the proportion of participants who developed hypertension was significantly higher in the high-TyG group than in the low-TyG group (65.3 vs. 50%, *P* = 0.004; Fig. 3). Although this result partly reflects the cut-off's classification ability, it also reinforces the consistency of the association observed in the continuous analyses. Furthermore, this stratification facilitated a more precise characterization of the adverse cardiometabolic profiles associated with elevated TyG indices. Indeed, the high-TyG group had significantly higher baseline BMI, waist circumference, and HOMA-IR than the low-TyG group, while no significant differences were detected for age, renal function, and CRP. BP was higher in the high-TyG group, with a statistically significant difference only in DBP (Table 2). Logistic regression analysis confirmed a significant positive association between high-TyG and the development of hypertension without adjustment (high-TyG vs. low-TyG, OR: 1.88, 95% CI: 1.22–2.89) and after adjustment for the main confounders (model including excess body weight: OR: 1.62, 95% CI: 1.01–2.61; model including abdominal obesity: OR: 1.63, 95% CI: 1.01–2.63). Likewise, the percentage of participants who developed hypertension was greater in the high-HOMA-IR group than in the low-HOMA-IR group (61.8 vs. 45.9%, *P* < 0.01) (Fig. 3). The High-HOMA-IR group had higher baseline BMI, waist circumference, SBP and DBP, and TyG than the low-HOMA-IR group, whereas no significant differences were detected for age, renal function, and CRP (Table 2). Nevertheless, despite logistic regression analysis showing a positive association between high-HOMA-IR and the development of hypertension in the unadjusted model (high-HOMA-IR vs. low-HOMA-IR, OR: 1.90, 95% CI: 1.32–2.74), the association was lost in the fully adjusted model (model including excess body weight, OR: 1.44, 95% CI: 0.96–2.17; model including abdominal obesity, OR: 1.48, 95% CI: 0.99–2.23).

**TABLE 2:** Baseline characteristics of the study participants stratified by the triglyceride-glucose index or the HOmeostatis Model Assessment of Insulin Resistance index threshold

*n* = 482	High-TyG (>4.91)	Low-TyG (≤4.91)	High-HOMA-IR (>1.82)	Low-HOMA-IR (≤1.82)
Number of participants	118	364	238	244
Age (years)	50.0 (6.5)	49.9 (6.8)	50.1 (6.4)	49.9 (7.0)
BMI (kg/m^2^)	27.2 (2.6)^a^	26.1 (2.8)	27.2 (2.7)^b^	25.6 (2.7)
Waist circumference (cm)	95.4 (6.5)^a^	91.9 (8.2)	95.1 (7.2)^b^	90.4 (8.0)
Systolic BP (mmHg)	121.3 (9.8)	119.5 (10.2)	121.2 (10.2)^b^	118.7 (9.9)
Diastolic BP (mmHg)	79.7 (6.5)^a^	78.3 (6.5)	79.9 (6.4)^b^	77.3 (6.4)
eGFR (ml/min/1.73 m^2^)^c^	96.3 (1.1)	99.1 (1.2)	97.4 (1.2)	99.5 (1.2)
HOMA-IR (units)^c^	2.2 (1.7)^a^	1.7 (1.6)	2.7 (1.4)^b^	1.2 (1.4)
TyG (units)	5.1 (0.2)^a^	4.6 (0.2)	4.8 (0.3)^b^	4.7 (0.2)
C-reactive protein (mg/l)^c^^,^^d^	1.2 (2.4)	1.1 (2.6)	1.2 (2.7)	1.0 (2.4)

Data are expressed as means (SD). BP, blood pressure; eGFR, estimated glomerular filtration rate; HOMA-IR, HOmeostatic Model Assessment of Insulin Resistance index; TyG, triglyceride-glucose index.

a High-TyG vs. low-TyG: *P* < 0.05.

b High-HOMA-IR vs. low-HOMA-IR: *P* < 0.05.

c Data expressed as geometric mean.

d *n* = 436.

## DISCUSSION

To our knowledge, this is the first study that has explored the shape of the association between TyG and the development of hypertension in a European cohort. In particular, the main findings indicate a positive and linear association between TyG and the risk of new-onset hypertension in a middle-aged sample of men from an unselected sample of male working population; TyG values higher than 4.91 were associated with an approximately two-fold increased risk of hypertension, independent of potential confounders, including age and anthropometric indices.

In this context, several epidemiological studies have found a positive association between TyG and hypertension [20,21]. However, few studies have prospectively examined the relationship between TyG and new-onset hypertension. Only one study, by Sánchez-Íñigo *et al.*, assessed this relationship in a European cohort [22], involving 3637 Spanish participants of both sexes. Over an 8-year follow-up period, a doubled risk of hypertension was observed in men in the highest TyG quintile but not in women. Our findings are substantially in agreement with this sex-specific association. However, their analysis did not evaluate the shape of the association, nor identified optimal TyG thresholds, and excluded adjustments for lipid-lowering therapy, important aspects that were specifically addressed in our study. The more robust methodological design of our study, incorporating spline analysis and ROC-based threshold estimation, enables a more nuanced assessment of the TyG–hypertension association within a European context. Conversely, most existing literature has focused on Asian populations [20,21]. Several cohort studies in Chinese adults have confirmed a linear and positive association between TyG and incident hypertension, even after adjusting for confounders such as obesity and inflammation markers. For instance, in a Chinese population of 4755 participants, including only middle-aged and elderly male and female individuals, there was a linear and positive association between TyG and new-onset hypertension after 5.2 years of follow-up, despite the low incidence of hypertension (19.7%) [27]. In addition, the analysis of 4686 Chinese individuals found a positive predictive role of TyG on the risk of hypertension after 9 years of follow-up, based on both sexes and a similar incidence of hypertension, but without exploring the shape of the association [28]. However, the magnitude of the association observed in our study was stronger, possibly reflecting differences in cardiometabolic and/or genetic profiles. In contrast, other studies, including those involving Chinese cohorts, reported weaker or borderline associations, once measures of obesity were considered, suggesting a potential mediating effect of adiposity [29,30]. In our analysis, adjustment for waist circumference or BMI did not attenuate these associations, thereby indicating an independent role of the TyG index.

Importantly, there are inconsistencies regarding sex differences. Tsai *et al.* [31], in a cohort of young and apparently healthy Taiwanese adults (*n* = 2448), found no significant association in men, while observing a stronger predictive power for TyG in women with stage 2 hypertension. Similarly, a study by Wang *et al.* (*n* = 3414) reported a predictive value only in female and elderly Chinese participants [32]. It is plausible that hormonal, behavioural, and body composition differences between men and women contribute to sex-specific effects [33,34]. For instance, women may exhibit greater vascular sensitivity to metabolic disturbances or differing fat distribution patterns. Thus, our results are not directly generalizable to women, and further studies are needed to explore these settings. Furthermore, these findings contrast with ours, which focused exclusively on men, due to other population differences (e.g. age, ethnicity, and duration of follow-up), the application of more stringent diagnostic criteria for hypertension (130/80 mmHg), and the use of a different statistical approach. Two recent meta-analyses further supported a positive association between TyG and hypertension risk [20,21]. In both cases, the highest TyG values were associated with a higher risk of new-onset hypertension. Only one study detected a nonlinear relationship by dose–response analysis [20], while the other study did not test the linearity of the association [21]. Of note, both meta-analyses reported high heterogeneity and potential publication bias, which may have limited the strength of the pooled estimates. Furthermore, differences in ethnicity, sex distribution, diagnostic thresholds, and TyG calculation methods may all contribute to these variations. Moreover, one also pooled studies with different designs (i.e. cross-sectional, retrospective, and prospective) and considered incorrect data from the studies [20]. Our findings support and extend this evidence by demonstrating a robust linear association between TyG and new-onset hypertension in a homogeneous European male cohort. Additionally, we identified an optimal TyG threshold (4.91) that stratified individuals at a higher cardiovascular risk. This cut-off is substantially in line with the TyG values associated with increased risk in Asian cohorts (typically >4.6) [20], despite population and methodological differences.

In this context, little data on the comparison between TyG and other insulin resistance expressions are available. Only one study also evaluated the role of HOMA-IR [30] and showed a nonsignificant association between this index and new-onset hypertension in the fully adjusted model, in addition to the borderline role of TyG. In agreement with this study, our data underscored a nonsignificant association between HOMA-IR and the development of hypertension. However, our analysis indicated a significant association between baseline TyG and new-onset hypertension and a different shape of the association for TyG and HOMA-IR (i.e. linear for TyG and nonlinear for HOMA-IR).

It is important to acknowledge that, although statistically significant, the discriminatory capacity of both indices in this cohort was limited by a relatively low AUC and the associated performance metrics. In particular, the sensitivity of the identified TyG threshold (>4.91) was only 30%, reflecting its limited ability to detect a substantial proportion of individuals at elevated risk of developing hypertension. While this low sensitivity was partially offset by the relatively high specificity, it may nevertheless constrain the practical applicability of the TyG index as a screening instrument.

Furthermore, the discriminatory performance of the two indices, as assessed through ROC curve analysis, was broadly comparable, with no statistically significant difference in the AUC values. Nonetheless, our results reinforce the robustness of the association between TyG and incident hypertension, regardless of whether the index was analysed as a categorical or continuous variable, and after adjustment for major confounding factors. Moreover, stratification according to the respective thresholds identified subgroups (i.e. high-TyG and high-HOMA-IR) characterized by markedly adverse cardiometabolic profiles, in line with previous evidence [35–37]. Notably, HOMA-IR was not significantly associated with new-onset hypertension in the fully adjusted models, whereas the TyG index was. The observed linear relationship between TyG and hypertension risk further supports the notion of a potentially more consistent and reliable predictive capacity. Hence, despite the acknowledged limitations, the TyG index may serve as a feasible and cost-effective adjunctive marker for early cardiometabolic risk stratification, particularly in settings where access to more complex or resource-intensive diagnostic assays may be limited.

The results of this study are strengthened by: the soundness of the results; the careful standardization of data collection at both examinations; the prospective design with a relatively long length of follow-up; the identification of an optimal cut-off point by ROC curve; the assessment of the shape of the association between the baseline TyG and the development of hypertension; the comparison of the predictive role of TyG and HOMA-IR (a widely used tool to evaluate insulin resistance) on new-onset hypertension; and finally, the independent role of TyG from the effect of therapy [38–41], as participants with antihypertensive therapy at baseline were excluded from the analysis, and the analyses confirmed the predictive role of TyG independently of lipid-lowering and glucose-lowering therapy at baseline.

Nevertheless, the study has some limitations: the participation of only white adult male individuals is a limitation of the study, in particular, the lack of women in our cohort limits the generalizability of our findings; indeed, evidence from previous studies has demonstrated potential sex-specific differences in the predictive utility of TyG, possibly due to hormonal and metabolic factors. Thus, further studies in women and more ethnically diverse populations are warranted to assess whether similar associations and cut-offs apply; the observational design of the study does not allow for the establishment of a causal relationship. Nevertheless, although the underlying pathophysiological mechanisms linking TyG to the development of hypertension have not been specifically explored, the longitudinal design allows the temporal sequence of events to be established, thereby strengthening the inference of a potential causal association; the lack of intermediate parameters measured during the 8-year follow-up period; and the possible influence of unmeasured variables cannot be entirely ruled out. In particular, dietary factors such as salt and potassium intake, which are both strongly associated with insulin resistance and the risk of hypertension [3,42], were not systematically assessed in the present study. This is a relevant limitation, as excessive dietary salt is known to contribute to arterial stiffness, enhance sympathetic activity, and increase salt sensitivity of BP, particularly in overweight or insulin resistance individuals [3,43,44]. Similarly, insufficient potassium intake has been linked to impaired insulin sensitivity and an elevated cardiovascular risk [42,45]. Nevertheless, considering the relatively stable dietary patterns within the population over time, we evaluated salt and potassium intake at follow-up. No significant differences were observed between individuals who developed hypertension and those who did not, nor between participants in the high-TyG and high-HOMA-IR groups and their respective counterparts (data not shown). Hence, these findings suggest that variations in these specific dietary components are unlikely to account for the reported associations. Future studies should include detailed dietary assessments to better elucidate the potential confounding or mediating effects of these nutritional factors.

In conclusion, the main findings of this study indicate that TyG is linearly and positively associated with new-onset hypertension in middle-aged men. In particular, TyG values higher than 4.91 were associated with an approximately doubled risk of new-onset hypertension, independent of the main potential confounders.

In contrast, the modest discriminatory capacity of the TyG index, reflected by its relatively low AUC and sensitivity, warrants caution when interpreting its applicability in clinical practice. Nonetheless, the findings demonstrate the robustness of the association between TyG and incident hypertension when analysed as both a categorical and a continuous variable, independent of major confounders. Moreover, the predictive performance of TyG appeared to be more consistent than that of HOMA-IR, a widely used index for insulin resistance assessment. Therefore, despite its limited sensitivity, and given that hypertension is a well established major risk factor for cardiovascular disease and a leading cause of premature mortality worldwide [1,2], these findings support the potential utility of the TyG index as a simple, cost-effective, and noninvasive adjunctive tool for the early stratification of cardiovascular risk.

Further research is warranted to validate these observations, enhance the predictive performance of TyG, assess its integration into multifactorial risk assessment models, and to explore the impact of therapeutic interventions.

## ACKNOWLEDGEMENTS

Funding: this research did not receive any specific grant from funding agencies in the public, commercial, or not-for-profit sectors.

Ethical approval: all procedures performed in studies involving human participants were in accordance with the ethical standards of the institutional and/or national research committee and with the Helsinki Declaration and its later amendments or comparable ethical standards.

Informed consent: informed consent was obtained from all individual participants included in the study.

### Conflicts of interest

There are no conflicts of interest.

## Supplementary Material

Supplemental Digital Content
